# EULAR Sjogren’s Syndrome Patient Reported Index (ESSPRI) and Other Patient-Reported Outcomes in the Assessment of Glandular Dysfunction in Primary Sjögren’s Syndrome

**DOI:** 10.3390/life13101991

**Published:** 2023-09-29

**Authors:** Hirut Yadeta Ture, Na Ri Kim, Eon Jeong Nam

**Affiliations:** Division of Rheumatology, Department of Internal Medicine, Kyungpook National University School of Medicine, Daegu 42113, Republic of Korea; danucharmy@gmail.com (H.Y.T.); nr2251@hanmail.net (N.R.K.)

**Keywords:** Sjogren’s syndrome, ESSPRI, patient-reported outcomes, ClinTrialsESSDAI, glandular dysfunction

## Abstract

The EULAR Sjögren’s Syndrome Disease Activity Index (ESSDAI), EULAR Sjogren’s Syndrome Patient Reported Index (ESSPRI), and other patient-reported outcomes (PROs), such as the visual analog scale (VAS) for symptoms and EULAR sicca score (ESS), are used to assess the disease activity of primary Sjögren’s syndrome (pSS). Recently, Clinical ESSDAI (ClinESSDAI) and Clinical Trials ESSDAI (ClinTrialsESSDAI) were developed for objective clinical disease activity indexes. However, the relationship of ClinESSDAI and ClinTrialsESSDAI with PROs as well as that between ESSPRI and other PROs and the objective parameters of glandular function in pSS have not been established. Herein, we investigated the correlation of ESSPRI and other PROs with the objective parameters of glandular function and the relationship of PROs with ClinESSDAI and ClinTrialsESSDAI in 66 patients with pSS. Correlations were calculated with Spearman’s correlation coefficient. ClinTrialsESSDAI was correlated with ESSPRI, dryness (ESSPRI-Dryness), fatigue, and pain domains of ESSPRI, VAS for oral dryness (oral-VAS), and patient’s global assessment. Although ESSPRI did not correlate with the objective parameters of glandular function, ESSPRI-Dryness, ESS, and oral- and ocular-VAS did. These results suggest that ESSPRI-Dryness, ESS, and VAS for symptoms, but not ESSPRI, reflect the glandular dysfunction and that ClinTrialsESSDAI correlates with PROs for dryness in pSS.

## 1. Introduction

Primary Sjögren’s syndrome (pSS) is a slowly progressing systemic autoimmune disease that affects 0.1–0.6% of the general adult population with a female-to-male ratio of at least 9:1 [1]. The exocrine glands are predominantly affected, leading to their dysfunction commonly manifesting as dry mouth and dry eyes [1,2]. Dryness features of pSS may present with oral, ocular, skin, airways, or vaginal dryness, among which ocular and oral dryness are commonly reported [3]. Specifically, patients prioritize dryness in terms of symptom relief [3].

Over the past two decades, significant efforts have been put into developing and validating effective outcome-measuring scores for pSS [3,4]. Patient-reported outcomes (PROs), such as the visual analog scale (VAS) for symptoms [5], EULAR Sicca Score (ESS) [3], and patient’s global assessment (PGA), have been widely used to evaluate the subjective symptoms of patients with pSS [6]. In addition, the EULAR Sjogren’s Syndrome Patient-Reported Index (ESSPRI) and EULAR Sjogren’s Syndrome Disease Activity Index (ESSDAI) are EULAR-validated outcomes measuring scores that measure the subjective and objective clinical disease activity of patients with pSS, respectively [3,7,8]. Recently, Clinical ESSDAI (ClinESSDAI), which does not include the biological domain, was developed for measuring a “true” clinical effect [7]. By contrast, Clinical Trials ESSDAI (ClinTrialsESSDAI) consists of the frequently active clinical domains of the ESSDAI [8]. Although the evaluation of patients’ symptoms with ESSPRI is complementary to the evaluation of systemic features with ESSDAI [9], the relationship between ClinESSDAI and ClinTrialsESSDAI with PROs is not well-established.

For the assessment of glandular function, ESSPRI and other PROs are easy to use, time effective, and can be performed in rheumatology clinics unlike the objective glandular measures [3,10]. In this era, as targeted biologics therapies for pSS are being attempted more than before [11], knowledge regarding the correlation between subjective symptoms and objective measures is needed more than ever to conveniently and effectively assess patients with pSS. Although ESSPRI and other PROs are commonly used to evaluate the patient’s symptoms, the correlation of ESSPRI and other PROs with the objective parameters of glandular function is not well-established. Although most studies showed that ESSPRI is well-correlated with other PROs and health-related quality of life [2,7,12] data concerning the correlations of both ESSPRI and other PROs with objective glandular measures are conflicting. Some studies showed a weak correlation between subjective dryness and objective parameters of glandular function [9], whereas other studies showed a good correlation [13]. However, in these studies, objective glandular assessments were mostly limited to Schirmer’s test and the salivary flow rate (SFR) [12,13].

Therefore, in this study, by including comprehensive and detailed studies of objective glandular function, we studied the correlation of ESSPRI and other PROs with objective glandular function measurements. In addition, the relationship between PROs and objective clinical activity measures was evaluated.

## 2. Patients and Methods

### 2.1. Patients

We enrolled 66 patients diagnosed with pSS and those who fulfilled the 2002 American–European Consensus Group classification criteria [14] and filled out the ESSPRI questionnaire between April 2015 and December 2020. All patients were female, and the mean age was 49.2 ± 11.3 years. The median duration of sicca symptoms was 4.0 (interquartile range [IQR], 2.0–7.0) years at enrollment. The procedures performed in this study were in accordance with the Declaration of Helsinki and approved by the Institutional Review Board (IRB) of Kyungpook National University Hospital (IRB 2015-09-035). Written informed consent was obtained from all the participants involved in this study. 

### 2.2. Variables and Measures

For assessing the disease activity, ESSPRI [4], ESS [3], ESSDAI [4], ClinESSDAI [7], and ClintrialsESSDAI [8] were measured using previous definitions or as previously reported. Patients with pSS were classified according to ESSDAI and ESSPRI based on the definition proposed by the EULAR Sjögren’s Task Force [15]. The disease activity levels by an ESSDAI were defined as follows: low (ESSDAI < 5), moderate (5 ≤ ESSDAI ≤ 13), and high disease activity (ESSDAI ≥ 14). In addition, the patient-acceptable symptom state estimate was defined as an ESSPRI < 5, so unsatisfactory symptom status was defined as an ESSPRI ≥ 5. Ocular and oral dryness were measured using a 10-cm VAS. The Ocular Surface Disease Index (OSDI) is a 12-item validated questionnaire that includes questions regarding ocular symptoms, vision-related function, and problems with environmental triggers, with a score range of 0 to 100 [16]. The OSDI is a valid and reliable instrument for measuring the severity of ocular dryness. PGA was also rated using VAS.

Laboratory investigation included the complete blood cell count and the evaluation of acute phase reactants, including the erythrocyte sedimentation rate (ESR) and C-reactive protein (CRP); anti-nuclear antibodies (ANAs); anti-Ro/La antibodies, and serum levels of immunoglobulin G (IgG) and complements C3 and C4. 

The unstimulated and stimulated whole SFR was determined using the spitting method as previously described by López-Jornet P et al. [17] with some modifications. Instead of the oral Schirmer’s test used by Lopez-Jornet P et al. [17], we used the spitting method to collect the stimulated SFR, and a 10-min collection was conducted for optimal salivary collection as the duration of collection affects the measurement [18]. Additionally, to minimize the effect of diurnal variation, afternoon collection was conducted as salivary flow is likely to reach its peak flow [19,20]. Participants were scheduled for saliva collection any time between 1:00 p.m. and 5:00 p.m. and were instructed to refrain from eating, drinking, smoking, and brushing their teeth for 2 h before the tests. Unstimulated whole saliva was collected in disposable 50-mL conical tubes for 15 min, with the participants in a sitting position with their heads slightly tilted forward and their eyes open. They were also instructed to minimize movement and not to swallow any saliva. For stimulation of saliva excretion, we used 4% citric acid because higher-concentration citric acid was reported to have a stronger and more sustained stimulatory effect [21,22]. Stimulated whole saliva was collected for 10 min after applying a 4% citric acid drop to the lateral borders of the tongue two times every five minutes.

To evaluate the function of major salivary glands, salivary gland scintigraphy (SGS) with ^99m^Tc-pertechnetate was performed in 64 of 66 (97.0%) patients. For quantifying the salivary gland uptake, circular regions of interest were manually drawn over the bilateral parotid gland (PG) and submandibular gland (SMG) on the anterior images. Additionally, a circular region of interest was drawn over the left temporal region for measuring the background activity. The uptake ratio (UR), and percentage excretion and ejection fraction (EF) of salivary glands were defined as follows [23]: PG-UR = P_20/_BG_20_; PG-EF (%) = 100 × [(P_20_ − BG_20_)/BG_20_ − (P_wo_ − BG_wo_)/BG_wo_]/[(P_20_ − BG_20_)/BG_20_]; SMG-UR = SM_20/_BG_20_ and SMG-EF (%) = 100 × [(SM_20_ − BG_20_)/BG_20_ − (SM_wo_ − BG_wo_)/BG_wo_]/[(SM_20_ − BG_20_)/BG_20_] (P_20_ = counts/pixel of PGs in 20-min images; SM_20_ = counts/pixel of SMGs in 20-min images; BG_20_ = counts/pixel of the background in 20-min images; P_wo_ = counts/pixel of PGs in post-washout images; SM_wo_ = counts/pixel of SMGs in post-washout images and BG_wo_ = counts/pixel of the background in post-washout images). Minor salivary gland (MSG) biopsies were conducted, and glandular inflammation was detected in 57 of 66 (86.4%) patients. MSG biopsy specimens were graded by a pathologist, with the focus score (FS) read blindly according to a standardized protocol [24].

For assessing lacrimal dysfunction, type I Schirmer’s test, ocular staining scoring (OSS), and fluorescein tear break-up time (tBUT) [25] were performed for each eye. 

### 2.3. Statistical Analysis

Continuous variables, representing a Gaussian distribution, are expressed as means ± standard deviation (SD), and the difference between the two groups was assessed using the Student’s *t*-test. If variables do not follow a Gaussian distribution, they are expressed as medians with IQRs (25–75 percentile), and the difference between the groups was assessed using the Mann–Whitney U test. Categorical variables were compared using the Chi-squared test or Fisher’s exact test where applicable. Bivariate correlations were determined using Spearman’s correlation coefficient. All statistical analyses were performed at a significance level of α = 0.05 on both sides using SPSS Statistics for Windows (Version 25.0; IBM, Armonk, NY, USA).

## 3. Results

### 3.1. Clinical and Laboratory Findings of Patients with pSS

The demographic and clinical characteristics of all patients are summarized in Table 1. The median ESSPRI score was 4.5 (3.5–5.8), and the percentage of patients with unsatisfactory symptom status, defined as ESSPRI ≥ 5 [15], was 48.5%. The median ESS score was 6.3 (5.1–7.7), and the median VAS scores for ocular dryness (ocular-VAS) and oral dryness (oral-VAS) were 6.0 (4.0–7.0) and 7.0 (5.0–8.0), respectively.

The median ESSDAI, ClinESSDAI, and ClinTrialsESSDAI scores were 2.0 (1.0–6.0), 2.0 (0.0–5.8), and 2.0 (0.0–4.0), respectively. The percentage of patients with moderate-to-high disease activity (ESSDAI ≥5) was 28.8%. The six most frequently active clinical ESSDAI domains, except the biological domain (59.4%, 38/64 patients), were articular (any activity, 22.7%), hematological (18.2%), glandular (12.1%), cutaneous (12.1%), and constitutional (10.6%) domains. One-third of the patients had never experienced systemic complications, whereas 33 (50.0%) and 33 (50.0%) had the presence of systemic manifestations at enrollment. Serum IgG levels were elevated in 59.4% of patients, and anti-Ro and -La antibodies were detected in 69.2% and 29.2% of patients, respectively. The ESR and CRP levels were elevated in 53% and 7.6% of patients, respectively.

Among the 66 patients, 28 took medications, including hydroxychloroquine (HCQ), cyclosporine, mycophenolate mofetil, and low doses of corticosteroid. Overall, 26 patients were on HCQ and 13 were on more than one drug. According to the status of medication, treated patients did not demonstrate any significant differences in the PROs, except for ESSPRI-Fatigue, and the objective parameters of lacrimal and salivary gland functions compared with untreated patients. However, treated patients showed significantly lower objective clinical disease activity index scores, including ESSDAI (*p* = 0.001), ClinESSDAI (*p* = 0.001), and ClinTrialsESSDAI (*p* = 0.006), FS (*p* = 0.046), and the proportion of patients with elevated ESR (*p* = 0.024; Table S1).

### 3.2. Correlation of PROs with Objective Clinical Disease Activity Indexes

**Figure 1:**
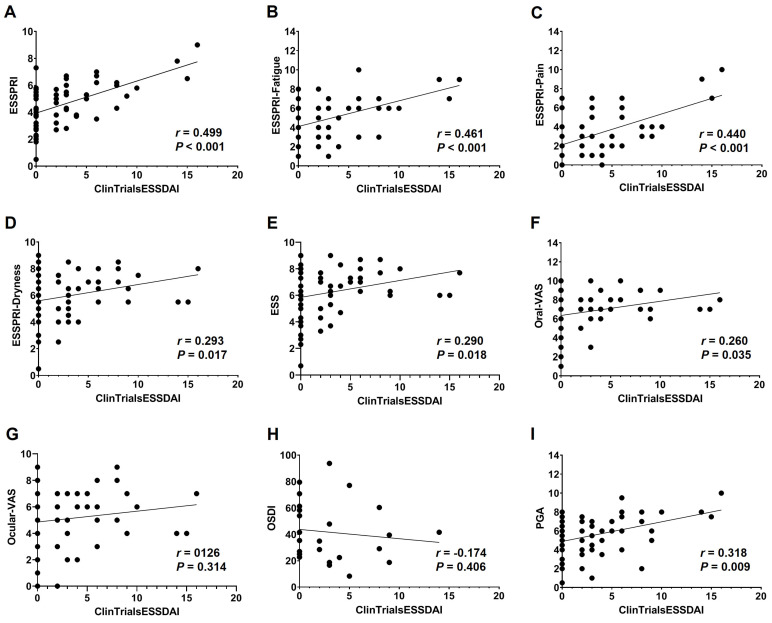
Correlations between Clinical Trial ESSDAI (ClinTrialsESSDAI) and subjective clinical disease activity indexes in patients with primary Sjögren’s syndrome. The correlation between ClinTrialsESSDAI and (**A**) ESSPRI, (**B**) ESSPRI-Fatigue, (**C**) ESSPRI-Pain, (**D**) ESSPRI-Dryness, (**E**) ESS, (**F**) oral-VAS, (**G**) ocular-VAS, (**H**) OSDI, and (**I**) PGA. ESSDAI, EULAR Sjögren’s Syndrome Disease Activity Index; ESSPRI, EULAR Sjögren’s Syndrome Patient Reported Index; ESSPRI-Fatigue, fatigue domain of ESSPRI; ESSPRI-Pain, pain domain of ESSPRI; ESSPRI-Dryness, dryness domain of ESSPRI; ESS, EULAR sicca score; Oral-VAS, visual analogue scale for oral dryness; Ocular-VAS, visual analogue scale for ocular dryness; OSDI, ocular surface disease index; PGA, patient’s global assessment.

The ESSPRI and its domains, except ESSPRI-Dryness, were significantly correlated with clinical disease activity indexes, including ESSDAI, ClinESSDAI, and ClinTrialsESSDAI, with the strongest correlation observed with ClinTrialsESSDAI (Table S2 and Figure 1). ESSPRI-Dryness, ESS, and oral-VAS were weakly but significantly correlated only with ClinTrialsESSDAI, whereas ocular-VAS and OSDI did not show any correlation with the objective clinical disease activity indexes. PGA showed a weak but significant correlation with ClinESSDAI and ClinTrialsESSDAI, but not ESSDAI. When categorized by treatment status, untreated patients showed a stronger correlation of ESSPRI, ESSPRI-Fatigue, ESSPRI-Pain, and PGA with ESSDAI, ClinESSDAI, and ClinTrialsESSDAI compared with the overall population; however, they showed no correlation between the PROs measuring dryness and the objective clinical disease activity indexes (Table S3). On the contrary, treated patients did not show any correlation between the subjective and objective clinical disease activity indexes.

### 3.3. Correlation between ESSPRI and Other PROs

ESSPRI was significantly correlated with other PROs, including ESS (*r* = 0.639, *p* < 0.001), ocular-VAS (*r* = 0.482, *p* < 0.001) and oral-VAS (*r* = 0.522, *p* < 0.001), and PGA (*r* = 0.673, *p* < 0.001; Table 2). Among the domains of ESSPRI, ESSPRI-Dryness was weakly correlated with ESSPRI-Fatigue (*r* = 0.360, *p* = 0.003) and ESSPRI-Pain (*r* = 0.376, *p* = 0.002). PGA was the most closely correlated with ESSPRI-Dryness (*r* = 0.690, *p* < 0.001) and the least closely correlated with ESSPRI-Pain (*r* = 0.409, *p* < 0.001; Table S4). ESSPRI-Dryness showed a high correlation with ESS (*r* = 0.969, *p* < 0.001), ocular-VAS (*r* = 0.799, *p* < 0.001), and oral-VAS (*r* = 0.811, *p* < 0.001).

ESS showed a high correlation with ocular-VAS (*r* = 0.657, *p* < 0.001), oral-VAS (*r* = 0.903, *p* < 0.001), and PGA (*r* = 0.674, *p* < 0.001). Although OSDI is a valid and reliable instrument for measuring the severity of ocular dryness [16], it was not significantly correlated with other PROs in the present study.

### 3.4. Correlation of PROs with the Objective Parameters of Glandular Function

The objective clinical disease activity indexes, including ESSDAI, ClinESSDAI, and ClinTrialsESSDAI, did not show any significant correlation with the objective measures of lacrimal and salivary gland function. ESSPRI and its domains, ESSPRI-Fatigue and ESSPRI-Pain, did not correlate with the objective parameters of glandular function (Table 3 and Table S4). Although patients with an unsatisfactory symptom status (ESSPRI ≥ 5) showed significant differences in PROs, except the OSDI, and objective clinical activity indexes, they did not show any significant differences in the objective parameters of glandular function compared with those with an ESSPRI of <5 (Table S5).

On the contrary, ESSPRI-Dryness showed a weak but statistically significant correlation with the objective parameters of salivary gland function, including the SFR (unstimulated: *r* = −0.281, *p* = 0.025; stimulated: *r* = −0.263, *p* = 0.037), SGS (PG-UR: *r* = −0.252, *p* = 0.044; SMG-UR: *r* = −0.357, *p* = 0.004; SMG-EF: *r* = −0.325, *p* = 0.009), and FS (*r* = 0.261, *p* = 0.050), and those of lacrimal gland function, including OSS (*r* = 0.333, *p* = 0.017) but not with tBUT (*r* = −0.220, *p* = 0.120), which showed a marginal significant correlation with PG-EF (*r* = −0.227, *p* = 0.071) and FS (*r* = 0.261, *p* = 0.050; Table 3). 

ESS tended to correlate more closely with the parameters of objective salivary gland function and OSS compared with ESSRI-Dryness, SFRs (unstimulated: *r* = −0.400, *p* = 0.001; stimulated: *r* = −0.364, *p* = 0.003), SGS (PG-UR: *r* = −0.325, *p* = 0.009; PG-EF: *r* = −0.285, *p* = 0.023; SMG-UR: *r* = −0.424, *p* < 0.001; SMG-EF: *r* = −0.356, *p* = 0.004), FS (*r* = 0.284, *p* = 0.032), and OSS (*r* = 0.305, *p* = 0.029; Table 3). When patients were divided into two groups based on the ESS score of 7, there were significant differences in the objective parameters of salivary gland function, including unstimulated (0.01 ± 0.00 vs. 0.04 ± 0.01, *p* = 0.023) and stimulated (0.22 ± 0.04 vs. 0.54 ± 0.11, *p* = 0.010) SFRs, SMG-UR (2.1 ± 0.8/0.1 vs. 2.6 ± 1.0/0.2, *p* = 0.016), and SMG-EF (13.2 ± 15.4/3.0 vs. 25.1 ± 18.2/3.0, *p* = 0.008) in the SGS examination between two groups (Table 4). However, objective lacrimal gland measures did not show any differences between the two groups.

In this study, the relationship between the subjective and objective measures of oral dryness was stronger than that between the subjective and objective measures of ocular dryness. Oral-VAS showed a significant correlation with the parameters of salivary gland function: SFRs (unstimulated: *r* = −0.527, *p* < 0.001; stimulated: *r* = −0.470, *p* < 0.001), SGS (PG-UR: *r* = −0.417, *p* = 0.001; PG-EF: *r* = −0.366, *p* = 0.003; SMG-UR: *r* = −0.467, *p* < 0.001; SMG-EF: *r* = −0.377, *p* = 0.002), and FS (*r* = 0.288, *p* = 0.030). For ocular dryness, ocular-VAS and OSDI were weakly correlated with OSS (ocular-VAS: *r* = 0.360, *p* = 0.009; OSDI: *r* = 0.414, *p* = 0.040) but not with tBUT (Table 3).

The objective parameters of lacrimal and salivary gland functions were not correlated with the patient’s age (Table S6). The objective parameters of salivary gland function were significantly correlated with each other, and OSS was also moderately significantly correlated with tBUT (*r* = −0.579, *p* < 0.001; Table 5). Additionally, OSS showed a significant correlation with the objective parameters of salivary gland function, except the unstimulated SFR, although tBUT was not correlated with any of the objective parameters of salivary gland function.

## 4. Discussion

In this study, although ESSPRI was significantly correlated with other PROs, including the ESS, ocular- and oral-VAS, and PGA, it did not correlate with the objective parameters of lacrimal and salivary glandular function. However, ESSPRI-Dryness, ESS, and ocular- and oral-VAS showed a significant correlation with the objective parameters of lacrimal and salivary glands function, except tBUT. In addition, ClinTrialsESSDAI but not ClinESSDAI showed a weak but significant correlation with the PROs, including ESSPRI, ESSPRI-Dryness, ESS, and oral-VAS.

Mucosal dryness due to exocrinopathy with inflammatory cell infiltration of the exocrine glands, especially lacrimal and salivary glands, is a key clinical feature of pSS, and its evaluation depends on both subjective PROs and objective glandular function measures [26]. However, the spectrum of clinical symptoms encompassed by the terms “dry eye” and “dry mouth” is wide and heterogeneous; therefore, PROs for dryness in pSS may be not closely correlated with the functional status of glandular secretion [26]. ESSPRI was developed by the ELUAR Sjögren’s Syndrome task force for evaluating patients’ symptoms [3]. In terms of the relationship between ESSPRI and objective parameters of glandular function, ESSPRI has only one domain on dryness, which refers to overall dryness and is not specific to ocular and oral dryness. Therefore, reflecting the objective function of salivary and lacrimal glands may be difficult with ESSPRI. Some studies demonstrated a lack of correlation between ESSPRI and a small number of exocrine functional measures such as both unstimulated and stimulated SFRs [13,27] or Schirmer’s test [13]. In this study, we performed a more diverse set of exocrine functional measures, including unstimulated and stimulated SFRs, SGS examination, and MSG biopsy as salivary gland function measures, and Schirmer’s test, OSS, and tBUT as lacrimal gland function measures. However, we could not find a significant correlation between the ESSPRI and the abovementioned objective glandular measures. Furthermore, patients with ESSPRI ≥5, that is, an unsatisfactory symptom status, did not show any differences in objective functional measures of salivary and lacrimal glands. On the contrary, ESSPRI was well-correlated with other PROs in this study. Furthermore, several previous studies also showed results that were consistent with our finding that a close correlation exists between ESSPRI and other PROs [3,4,7]. ESSPRI is composed of a VAS for pain and fatigue as well as a VAS for general dryness [3]; therefore, its significant correlation with other PROs is a plausible finding [3,4,7]. 

Unlike ESSPRI, ESSPRI-Dryness, ESS, ocular- and oral-VAS, and OSDI focus more on the subjective symptoms of dryness in patients with pSS. The majority of complaints made by patients with pSS include ocular and oral dryness. ESSPRI-Dryness is a domain of ESSPRI that assesses the overall dryness of the entire body without specifying the site [3,13]. Therefore, conflicting results have been reported on the correlation of ESSPRI-Dryness with the objective parameters of lacrimal or salivary gland function. Hijjaw et al. demonstrated a significant correlation of ESSPRI-Dryness with the salivary gland functional measure using the unstimulated SFR [27], whereas Lackner et al. used the Schirmer’s test and unstimulated and stimulated SFRs to argue that there is no correlation between ESSPRI-Dryness and glandular function measures [13]. In this study, we performed a more diverse set of measures for salivary and lacrimal gland functions and showed that there is a low but significant correlation of ESSPRI-Dryness with unstimulated and stimulated SFRs, SGS, and OSS. 

ESS is a PRO that only considers oral and ocular dryness [3]; therefore, ESS may tend to correlate better with the objective measures of salivary and lacrimal gland function than ESSPRI-Dryness. Ocular- and oral-VAS assess only symptoms of the lacrimal and salivary glands, respectively, and therefore, may better reflect the degree of exocrine function of salivary and lacrimal glands than other PROs. Therefore, ESS and ocular- and oral-VAS may better reflect the functional status of lacrimal and salivary glands than ESSPRI or ESSPRI-Dryness. In this study, ESSPRI-Dryness, ESS, and ocular- and oral-VAS showed a significant correlation with objective glandular measures. Among these, ESSPRI-Dryness reflected the glandular function the least and ocular- and oral-VAS were the most closely correlated with the glandular measures. In addition, when patients were divided into two groups based on the ESS score of 7, there were significant differences in the objective parameters of salivary gland function between the two groups, unlike those based on an ESSPRI score of 5.

The relationship between the subjective and objective measures of ocular dryness appears to be weaker than the subjective and objective measures of oral dryness [11,22,24]. Several studies demonstrated a weak but significant correlation between the objective and subjective measures of ocular dryness [28,29,30]. For example, Vehof et al. showed that, among the several dry eye tests, only corneal and conjunctival staining scores were somewhat indicative of the severity of dry eye symptoms [28]. We also found that the correlation of PROs with the objective lacrimal gland measures was weaker than that of PROs with the objective salivary gland measures. OSS showed a weak correlation with the PROs, including ESSPRI-Dryness, ESS, Ocular-VAS, and OSDI, and tBUT was not correlated with any PROs in this study. On the contrary, some studies showed an inverse correlation between ocular symptom severity and objective measures, which may be attributed to a decreased corneal sensation resulting from sensory nerve damage in patients with dry eye [31,32]. This may be a part of the explanation for why the subjective and objective ocular dryness measures correlate relatively weakly [33]. 

Ocular dryness in pSS can be because of either a deficiency of aqueous secretion, an impairment of the lipid layer, an impairment of the mucin gel, or a combination of the three [34]. The diagnostic tests used to assess patients with dry eye symptoms are as follows: Schirmer’s test, which quantitatively measures the tear production; epithelial staining with vital dyes such as Rose Bengal, lissamine green, and fluorescein, which explores the presence of punctate epitheliopathy; tBUT, which evaluates the stability of the tear film by measuring the time required for the tear film to break up following a blink and tear [26,35]. However, these objective measures have some limitations [35]. Scoring of staining of punctate epitheliopathy may fluctuate over the day, and these scales are more difficult to apply to atypical staining patterns. Fluorescein tBUT is highly influenced by environmental factors such as room temperature, air conditioning, and the operator’s concentration. A tiny break-up in the tears can be easily missed. Furthermore, both tBUT and OSS are easily affected by the amount of fluorescein instilled and the amount of tear volume in the eye can vary between patients. Therefore, depending on which glandular function measures are performed and to what extent, there may be differences in the degree to which these PROs reflect the objective lacrimal gland measure.

To have a complete “picture” of SS, it is recommended to evaluate patients using both a disease activity instrument, such as ESSDAI, and a PRO, such as ESSPRI [12]. Previous studies showed that ESSPRI and ESSDAI were not correlated with each other but have complementary roles in the assessment of disease activity in patients with pSS because they measure different aspects of the disease [2,11,32]. Interestingly, contrary to previous studies, we found a weak but significant correlation between ESSPRI and its domains, except ESSPRI-Dryness, with the objective clinical disease activity indexes, including ESSDAI, ClinESSDAI, and ClinTrialsESSDAI, with the strongest correlation observed with ClinTrialsESSDAI. ClinTrialsESSDAI, unlike ESSDAI and ClinESSDAI, also showed a very weak significant correlation with PROs for dryness, including ESSPRI-Dryness, ESS, and oral-VAS. Seror et al. [9] showed that ESSPRI was significantly correlated with SSDAI [36] and Sjögren’s Systemic Clinical Activity Index [36], which are objective clinical activity indexes with subjective items. ESSDAI also has a constitutional domain, and 10.6% of patients in this study had an active constitutional domain. In addition, ClinTrialsESSDAI consists of six frequently active clinical domains of ESSDAI [8] and has a large overlap with the eight items of SSDAI [36]. These are the likely reasons why objective clinical disease activity indexes were correlated with PROs and why ClinTrialsESSDAI was the most closely correlated with ESSPRI in this study. Furthermore, we reanalyzed the correlation on the base of the status of medication intake and found a significant correlation between the subjective and objective clinical disease activity indexes only in untreated patients; however, treated patients did not show any correlation between them. This result may be related to the results that treatment improved ESSDAI but not ESSPRI [37]. In this study, when compared according to the medication status, treated patients demonstrated a significant difference in objective clinical disease activity indexes and FS, whereas they did not show any significant differences in the PROs, except ESSPRI-Fatigue. 

It is worth noting that the process of salivary collection is a delicate procedure that is affected by various subtle environmental and interpersonal variations [20]. Basal SFR is affected by diurnal and seasonal variations, the hydration status of the patient, the collection environment, and the body position during collection [20,38]. Additionally, the nature, duration, and intensity of the stimulus also affect the salivary flow [18]. In our study, we have tried to minimize the effect of those factors by using a higher concentration of citric acid, that is 4%, according to Lopez-Jornet P et al. with modification. Although the duration of sample collection varies among the previous publications [38,39,40], we collected a stimulated SFR for 10 min. We also tried to minimize the diurnal variation by adjusting the time of collection to the afternoon as the peak usually is reached during this time of the day [19,20,38].

This study has some limitations. First, we did not perform the salivary gland ultrasound (SGUS) for the objective salivary gland measure. SGUS is a well-tolerated, noninvasive, non-irradiating, popular diagnostic method for assessing the involvement of major salivary glands in pSS [41] and may represent a good option as a first-line imaging tool in the diagnostics of the disease [42]. Although SGS was excluded from the later classification criteria for pSS owing to its low specificity to pSS [43], its results correlated with the clinical and histopathological features of salivary glands in patients with SS [14,44]. In addition, a recent study showed that SGUS scores for the PG and SMG were significantly correlated with PG-UR and PG-EF as well as SMG-UR and SMG-EF, and patients with pSS who presented with definite abnormal findings on SGUS showed more salivary gland dysfunction evaluated using SGS [45]. Second, this was a single-center cross-sectional study with a limited sample size. For consistent results, multicenter studies are required. Additionally, longitudinal studies may also be needed to see whether these correlations change over time and to determine the factors affecting these correlations. Third, all participants in our study were females. One reason for the ‘all-female’ data could be the higher female-to-male ratio of pSS in Asian patients than what has been commonly reported which is 9:1 [46,47]. In one large cross-sectional study, the highest female-to-male ratio of as high as 27:1 was reported in Asians while the lowest ratio was reported in Blacks, at 7:1 [46]. In a nationwide, population-based study in South Korea, the female-to-male ratio was 14.5:1 among 5891 patients newly diagnosed with pSS between 2010 and 2014 [47]. Additionally, the smaller size of our study population might have been a contributing factor. Similar to our case, in another study of pSS patients from Korea, all 104 participants were female [39]. 

## 5. Conclusions

In pSS, the unestablished correlation between PROs and objective glandular functions has been one of the unanswered questions. The results of this study indicated that PROs, except ESSPRI, ESSPRI-Fatigue, and ESSPRI-Pain, were significantly correlated with the objective gland measures, which may imply that they can reflect the glandular function. In addition, ClinTrialsESSDAI showed a significant correlation with ESSPRI and PROs for dryness, including ESSPRI-Dryness, ESS, and oral-VAS. This has great implications, especially during the follow-up of patients with pSS. Although a smaller sample size was a limitation of this study, it provides insights into the topic and encourages future larger and multicenter studies.

## Figures and Tables

**Table 1:** Demographic and clinical characteristics of patients with primary Sjögren’s syndrome.

	Primary Sjögren’s Syndrome (N = 66)
Age, years	49.2 ± 11.3
Disease duration, years	4.0 (2.0–7.0)
Sex, female (%)	66 (100)
Patient-reported outcomes (PROs)	
ESSPRI	4.5 (3.5–5.8)
ESSPRI-Dryness	6.0 (5.0–7.0)
ESSPRI-Fatigue	5.0 (3.0–6.0)
ESSPRI-Pain	3.0 (1.0–4.0)
ESS	6.3 (5.1–7.7)
Ocular-VAS	6.0 (4.0–7.0)
Oral-VAS	7.0 (5.0–8.0)
OSDI (N = 25)	35.4 (25.0–58.3)
PGA	6.0 (4.0–7.0)
Objective Clinical Disease Activity Indexes	
ESSDAI	2.0 (1.0–6.0)
ClinESSDAI	2.0 (0.0–5.8)
ClinTrialsESSDAI	2.0 (0.0–4.0)
Lacrimal gland function measures	
Schirmer’s test ≤5 mm/5 min	52 (78.8)
OSS (N = 51)	5.0 (2.0–10.0)
OSS ≥4	33/51 (64.7)
tBUT (N = 51)	8.0 (6.0–11.0)
Salivary gland function measures	
Unstimulated SFR, mL/min	0.01 (0.00–0.04)
Stimulated SFR, mL/min	0.22 (0.11–0.47)
Salivary gland scintigraphy (N = 64)	
PG-UR	2.5 (1.7–3.1)
PG-EF (%)	40.7 (21.8–55.1)
SMG-UR	2.1 (1.8–2.5)
SMG-EF (%)	14.5 (6.3–32.4)
Minor salivary gland biopsy (N = 57)	
Focus score	1.6 (0.8–2.5)
Laboratory results	
ANA	59/66 (89.4)
Anti-SSA/Ro	45/65 (69.2)
Anti-SSB/La	19/65 (29.2)
IgG, mg/dL (N = 64)	1747.5 (1362.0–2032.3)
IgG elevation (>1600 mg/dL)	38/64 (59.4)
ESR, mm/h	22.0 (10.5–34.0)
ESR elevation (>20 mg/h)	35 (53.0)
CRP, mg/dL	0.1 (0.1–0.1)
CRP elevation (>0.5 mg/dL)	5 (7.6)
Complement C3 (mg/dL)	100.7 (84.8–112.6)
Complement C4 (mg/dL)	22.7 (17.5–28.0)
Current medications	28 (42.4)
Steroid	1 (1.5)
MMF	4 (6.1)
AZA	7 (10.6)
CsA	4 (6.1)
HCQ	26 (39.4)

Data are described as means ± standard deviation, medians with the interquartile range, or number of patients (%). ESSPRI, European League Against Rheumatism EULAR Sjögren’s Syndrome Patient Reported Index; ESSPRI-Dryness, dryness domain of ESSPRI; ESSPRI-Fatigue, fatigue domain of ESSPRI; ESSPRI-Pain, pain domain of ESSPRI; ESS, EULAR sicca score; Ocular-VAS, visual analog scale for ocular dryness; Oral-VAS, visual analog scale for oral dryness; OSDI, ocular surface disease index; PGA, patient’s global assessment; ESSDAI, EULAR Sjögren’s Syndrome Disease Activity Index; ClinESSDAI, Clinical ESSDAI; ClinTrialsESSDAI, Clinical Trials ESSDAI; OSS, ocular staining score; tBUT, tear break-up time; SFR, salivary flow rate; PG, parotid gland; UR, uptake ratio; EF, ejection fraction; SMG, submandibular gland; ANA, anti-nuclear antibody; ESR, erythrocyte sedimentation rate; CRP, C-reactive protein; MMF, mycophenolate mofetil; AZA, azathioprine; CsA, cyclosporine; HCQ, hydroxychloroquine.

**Table 2:** Correlations between subjective clinical activity indexes in patients with primary Sjögren’s syndrome.

	ESSPRI		ESSPRI-Dryness		ESS		Ocular-VAS		Oral-VAS	
	Spearman Rho	*p*-Value	Spearman Rho	*p*-Value	Spearman Rho	*p*-Value	Spearman Rho	*p*-Value	Spearman Rho	*p*-Value
ESSPRI	-	-	0.661	<0.001	0.639	0.001	0.482	<0.001	0.522	<0.001
ESSPRI-Dryness	0.661	<0.001	-	-	0.969	<0.001	0.811	<0.001	0.799	<0.001
ESSPRI-Fatigue	0.859	<0.001	0.360	0.003	0.324	0.008	0.289	0.019	0.241	0.052
ESSPRI-Pain	0.849	<0.001	0.376	0.002	0.368	0.002	0.208	0.094	0.328	0.007
ESS	0.639	<0.001	0.969	<0.001	-	-	0.657	<0.001	0.903	<0.001
Ocular-VAS	0.482	<0.001	0.799	<0.001	0.657	<0.001	-	-	0.297	0.015
Oral-VAS	0.522	<0.001	0.811	<0.001	0.903	<0.001	0.297	0.015	-	-
OSDI	0.162	0.440	0.272	0.189	0.229	0.270	0.296	0.189	0.195	0.351
PGA	0.673	<0.001	0.690	<0.001	0.674	<0.001	0.527	<0.001	0.575	<0.001

ESSPRI, EULAR Sjögren’s Syndrome Patient Reported Index; ESSPRI-Dryness, dryness domain of ESSPRI; ESSPRI-Fatigue, fatigue domain of ESSPRI; ESSPRI-Pain, pain domain of ESSPRI; ESS, EULAR sicca score; Ocular-VAS, visual analog scale for ocular dryness; Oral-VAS, visual analog scale for oral dryness; OSDI, ocular surface disease index; PGA, patient’s global assessment.

**Table 3:** Correlations between the subjective clinical activity indexes and the objective parameters of gland functions in patients with primary Sjögren’s syndrome.

	ESSPRI		ESSPRI-Dryness		ESS		Ocular-VAS		Oral-VAS	
	Spearman Rho	*p*-Value	Spearman Rho	*p*-Value	Spearman Rho	*p*-Value	Spearman Rho	*p*-Value	Spearman Rho	*p*-Value
OSS	−0.009	0.950	0.333	0.017	0.305	0.029	0.360	0.009	0.211	0.138
t-BUT	−0.005	0.973	−0.220	0.120	−0.219	0.122	−0.213	0.134	−0.161	0.259
Unstimulated SFR	−0.083	0.518	−0.281	0.025	−0.400	0.001	0.036	0.779	−0.527	<0.001
Stimulated SFR	−0.042	0.746	−0.263	0.037	−0.364	0.003	0.032	0.806	−0.470	<0.001
Salivary gland scintigraphy										
PG-UR	−0.083	0.516	−0.252	0.044	−0.325	0.009	−0.005	0.969	−0.417	0.001
PG-EF (%)	−0.143	0.260	−0.227	0.071	−0.285	0.023	−0.001	0.996	−0.366	0.003
SMG-UR	−0.001	0.991	−0.357	0.004	−0.424	<0.001	−0.148	0.245	−0.467	<0.001
SMG-EF (%)	−0.123	0.333	−0.325	0.009	−0.356	0.004	−0.155	0.221	−0.377	0.002
Focus score	0.133	0.325	0.261	0.050	0.284	0.032	0.104	0.443	0.288	0.030
ESR	0.271	0.028	0.156	0.210	0.148	0.234	0.130	0.297	0.076	0.543
CRP	0.322	0.008	0.133	0.289	0.128	0.304	0.131	0.293	0.032	0.800
IgG	0.266	0.033	0.209	0.097	0.214	0.090	0.235	0.062	0.079	0.533

ESSPRI, EULAR Sjögren’s Syndrome Patient Reported Index; ESSPRI-Dryness, dryness domain of ESSPRI; ESS, EULAR sicca score; Ocular-VAS, visual analog scale for ocular dryness; Oral-VAS, visual analog scale for oral dryness; OSS, ocular staining score; tBUT, tear break-up time; SFR, salivary flow rate; PG, parotid gland; UR, uptake ratio; EF, ejection fraction; SMG, submandibular gland; ESR, erythrocyte sedimentation rate; CRP, C-reactive protein.

**Table 4:** Clinical and laboratory characteristics of patients with primary Sjögren’s syndrome based on the EULAR Sicca Score of 7.

	ESS ≥ 7 (N = 27)			ESS < 7 (N = 39)			*p*-Value
Age, years	50.6	±	11.0	48.3	±	11.6	0.427 ^†^
Disease duration, years	4.4	±	2.5	5.6	±	4.1	0.160
ESSPRI	5.6	±	1.3	3.9	±	1.5	<0.001
ESSPRI-Dryness	7.5	±	0.7	4.8	±	1.3	<0.001
ESSPRI-Fatigue	5.6	±	2.0	4.4	±	2.1	0.020
ESSPRI-Pain	3.7	±	2.2	2.6	±	2.3	0.061
Ocular-VAS	7.5	±	0.7	4.8	±	1.3	<0.001
Oral-VAS	8.5	±	0.9	5.6	±	1.9	<0.001
OSDI	50.0	±	28.9	37.0	±	17.1	0.168
PGA	6.8	±	1.5	4.6	±	1.9	<0.001
ESSDAI	5.1	±	5.6	3.5	±	4.7	0.197
ClinESSDAI	4.7	±	5.9	3.2	±	5.6	0.277
ClinTrialsESSDAI	3.7	±	4.0	2.3	±	3.7	0.148
Schirmer’s test positivity	23/26		(88.5)	29/39		(74.4)	0.214 ^‡^
OSS	8.2	±	6.8	5.7	±	5.6	0.161
tBUT	7.8	±	3.0	9.9	±	4.9	0.082
Unstimulated SFR (mL/min)	0.01	±	0.02	0.04	±	0.07	0.023
Stimulated SFR (mL/min)	0.22	±	0.21	0.54	±	0.65	0.010
Salivary gland scan							
PG-UR	2.2	±	0.9	2.7	±	0.9	0.066
PG-EF (%)	32.1	±	21.5	41.8	±	20.4	0.073
SMG-UR	2.1	±	0.8	2.6	±	1.0	0.016
SMG-EF (%)	13.2	±	15.4	25.1	±	18.2	0.008
Focus score	2.4	±	1.9	1.7	±	1.8	0.161
ESR elevation	15/27		(55.6)	20/39		(51.3)	0.805 ^‡^
CRP elevation	2/27		(7.4)	3/39		(7.7)	1.000 ^‡^
IgG elevation	17/27		(63.0)	21/37		(56.8)	0.797 ^‡^
Anti-Ro antibody positivity	20/26		(76.9)	25/39		(64.1)	0.441 ^‡^
Anti-La antibody positivity	7/26		(26.9)	12/39		(30.8)	0.787 ^‡^

*p*-values are obtained using the Student’s *t*-test ^†^, Mann–Whitney U test, or Chi-squared or Fisher’s exact test ^‡^. ESS, EULAR sicca score; ESSPRI, EULAR Sjögren’s Syndrome Patient Reported Index; ESSPRI-Dryness, dryness domain of ESSPRI; ESSPRI-Fatigue, fatigue domain of ESSPRI; ESSPRI-Pain, pain domain of ESSPRI; Ocular-VAS, visual analog scale for ocular dryness; Oral-VAS, VAS for oral dryness; OSDI, ocular surface disease index; PGA, patient’s global assessment; ESSDAI, EULAR Sjögren’s Syndrome Disease Activity Index; ClinESSDAI, Clinical ESSDAI; ClinTrialsESSDAI, Clinical Trials ESSDAI; OSS, ocular staining score; tBUT, tear break-up time; SFR, salivary flow rate; PG, parotid gland; UR, uptake ratio; EF, ejection fraction; SMG, submandibular gland; ESR, erythrocyte sedimentation rate; CRP, C-reactive protein.

**Table 5:** Correlation between the objective parameters of lacrimal and salivary gland function in patients with primary Sjögren’s syndrome.

		Stimulated SFR	PG-UR	PG-EF (%)	SMG-UR	SMG-EF (%)	Focus Score	OSS	tBUT
Unstimulated SFR	Spearman rho	0.659	0.467	0.431	0.364	0.338	−0.195	−0.221	0.146
	*p*-value	<0.000	0.000	0.001	0.004	0.008	0.158	0.131	0.323
Stimulated SFR	Spearman rho	1.000	0.745	0.669	0.661	0.489	−0.206	−0.453	0.248
	*p*-value		<0.000	0.000	0.000	0.000	0.135	0.001	0.089
PG-UR	Spearman rho		1.000	0.702	0.653	0.507	−0.238	−0.476	0.160
	*p*-value			<0.000	<0.000	<0.000	0.080	<0.000	0.268
PG-EF (%)	Spearman rho			1.000	0.402	0.615	−0.291	−0.362	0.252
	*p*-value				<0.001	<0.000	0.031	0.010	0.078
SMG-UR	Spearman rho				1.000	0.603	−0.273	−0.378	0.162
	*p*-value					<0.000	0.044	0.007	0.262
SMG-EF (%)	Spearman rho					1.000	−0.234	−0.287	0.108
	*p*-value						0.086	0.043	0.455
Focus score	Spearman rho						1.000	0.362	−0.219
	*p*-value							0.013	0.144
OSS	Spearman rho							1.000	−0.579
	*p*-value								<0.000

SFR, salivary flow rate; PG, parotid gland; UR, uptake ratio; EF, ejection fraction; SMG, submandibular gland; OSS, ocular staining score; tBUT, tear break-up time.

## Data Availability

Data are contained within this article.

## Supplementary Materials

The following supporting information can be downloaded at: https://www.mdpi.com/article/10.3390/life13101991/s1, Table S1: Clinical and laboratory characteristics of patients with primary Sjögren’s syndrome according to the status of medication; Table S2: Correlations between subjective and objective clinical disease activity indexes in patients with primary Sjögren’s syndrome; Table S3: Correlations between subjective and objective clinical disease activity indexes according to status of medication in primary Sjögren’s syndrome; Table S4: Correlations between domains of ESSPRI and other PROs or objective clinical disease activity indexes in patients with primary Sjögren’s syndrome; Table S5: Clinical and laboratory characteristics of patients with primary Sjögren’s syndrome based on ESSPRI of 5; Table S6: Correlations between age or disease duration and subjective or objective measures in primary Sjögren’s syndrome.

## Abbreviations

ESSDAI	European League Against Rheumatism (EULAR) Sjögren’s Syndrome Disease Activity Index
ClinESSDAI	Clinical EULAR Sjögren’s Syndrome Disease Activity Index
ClinTrialsESSDAI	Clinical Trials EULAR Sjögren’s Syndrome Disease Activity Index
ESSPRI	EULAR Sjögren’s Syndrome Patient Reported Index
ESSPRI-Dryness	Dryness domain of EULAR Sjögren’s Syndrome Patient Reported Index
ESSPRI-Fatigue	Fatigue domain of EULAR Sjögren’s Syndrome Patient Reported Index
ESSPRI-Pain	Pain domain of EULAR Sjögren’s Syndrome Patient Reported Index
ESS	EULAR sicca score
Ocular-VAS	Visual analogue scale for ocular dryness
Oral-VAS	Visual analogue scale for oral dryness
OSDI	Ocular surface disease index
PGA	Patient’s global assessment
OSS	Ocular staining score
tBUT	Tear break-up time
SFR	Salivary flow rate
SGS	Salivary gland scan
SGUS	Salivary gland ultrasound
PG	Parotid gland
SMG	Submandibular gland
UR	Uptake ratio
EF	Ejection fraction
FS	Focus score
ANA	Anti-nuclear antibody
