# Supplementary material for: EULAR Sjogren’s Syndrome Patient Reported Index (ESSPRI) and Other Patient-Reported Outcomes in the Assessment of Glandular Dysfunction in Primary Sjögren’s Syndrome
Source: Life (Basel). 2023 Sep 29;13(10):1991. doi: 10.3390/life13101991 (PMC10608572; doi:10.3390/life13101991)
Supplement: Supplementary file 1 [file life-13-01991-s001.zip › life-2604853-supplementary.pdf]

Supplementary Table S1. Clinical and laboratorial characteristics of patients with primary Sjögren's syndrome according to the status of medication.

	Medication (+) (N = 28)	Medication (-) (N = 38)	*P*-value
Age, years	51.3 ± 10.5	47.7 ± 11.8	0.212†
Disease duration	7.1 ± 3.9	3.6 ± 2.4	<0.001
ESSPRI	4.2 ± 1.4	4.9 ± 1.8	0.088
ESSPRI-Dryness	5.7 ± 1.4	6.1 ± 2.0	0.441
ESSPRI-Fatigue	4.2 ± 2.0	5.3 ± 2.1	0.033
ESSPRI-Pain	2.7 ± 2.1	3.3 ± 2.4	0.290
ESS	5.9 ± 1.5	6.4 ± 1.9	0.223
Ocular-VAS	5.3 ± 1.6	5.0 ± 2.5	0.588
Oral-VAS	6.2 ± 2.1	7.2 ± 2.1	0.070
OSDI	43.7 ± 22.0	40.7 ± 23.3	0.918
PGA	5.3 ± 1.9	5.7 ± 2.2	0.431
ESSDAI	2.1 ± 2.2	5.7 ± 6.0	0.001
ClinESSDAI	1.5 ± 2.0	5.6 ± 6.8	0.001
ClintrialsESSDAI	1.5 ± 2.0	3.8 ± 4.6	0.006
Schirmer' test positivity	22/28 (78.6)	30/37 (81.1)	1.000‡
OSS	7.2 ± 5.6	6.8 ± 6.6	0.829
tBUT	8.6 ± 3.7	9.0 ± 4.5	0.746
Unstimulated SFR (ml/min)	0.03 ± 0.05	0.03 ± 0.06	0.734
Stimulated SFR (ml/min)	0.35 ± 0.41	0.45 ± 0.61	0.461
Salivary gland scintigraphy			
PG-UR	2.4 ± 0.8	2.6 ± 1.0	0.373
PG-EF (%)	35.8 ± 23.2	39.4 ± 19.9	0.502
SMG-UR	2.4 ± 1.0	2.4 ± 0.9	0.705
SMG-EF (%)	19.7 ± 18.6	20.7 ± 17.8	0.843
Focus score	1.4 ± 1.3	2.4 ± 2.0	0.046
ESR elevation	10/28 (35.7)	25/38 (65.8)	0.024‡
CRP elevation	1/28 (3.6)	4/38 (10.5)	0.385‡
IgG elevation	13/26 (50.0)	25/38 (65.8)	0.300‡
Anti-Ro antibody positivity	20/28 (71.4)	25/37 (67.6)	0.792‡
Anti-La antibody positivity	8/28 (28.6)	11/37 (29.7)	1.000‡

Data are described as the mean ± standard deviation (SD), or number of patients (%). *P* values are obtained using the Student's *t*-test†, Mann–Whitney U test, or Chi-squared or Fisher's exact test‡. ESSPRI, EULAR Sjögren's Syndrome Patient Reported Index; ESSPRI-Dryness, dryness domain of ESSPRI; ESSPRI-Fatigue, fatigue domain of ESSPRI; ESSPRI-Pain, pain domain of ESSPRI; ESS, EULAR sicca score; Ocular-VAS, visual analogue scale for ocular dryness; Oral-VAS; VAS for oral dryness; OSDI, ocular surface disease Index; PGA, patient's global assessment; ESSDAI, EULAR Sjögren's Syndrome Disease Activity Index; ClinESSDAI, Clinical ESSDAI; ClintrialsESSDAI, Clinical Trials ESSDAI ; OSS, ocular staining score; tBUT, tear Break-up time; SFR, salivary flow rate; PG, parotid gland; UR, uptake ratio; EF, ejection fraction; SMG, submandibular gland, ESR, erythrocyte sedimentation rate; CRP, C-reactive protein.

Supplementary Table S2. Correlations between subjective and objective clinical disease activity indexes in patients with primary Sjögren's syndrome.

	ESSDAI		ClinESSDAI	
	**Spearman rho**	***P*-value**	**Spearman rho**	***P*-value**
ESSPRI	0.371	0.002	0.412	0.001
ESSPRI-Dryness	0.160	0.198	0.193	0.121
ESSPRI-Fatigue	0.395	0.001	0.388	0.001
ESSPRI-Pain	0.310	0.011	0.383	0.001
ESS	0.170	0.172	0.193	0.120
Ocular-VAS	0.030	0.814	0.019	0.877
Oral-VAS	0.181	0.145	0.178	0.152
OSDI	-0.160	0.444	-0.174	0.406
PGA	0.231	0.062	0.245	0.048

ESSDAI, EULAR Sjögren's Syndrome Disease Activity Index; ClinESSDAI, Clinical ESSDAI; ESSPRI, EULAR Sjögren's Syndrome Patient Reported Index; ESSPRI-Dryness, dryness domain of ESSPRI; ESSPRI-Fatigue, fatigue domain of ESSPRI; ESSPRI-Pain, pain domain of ESSPRI; ESS, EULAR sicca score; Ocular-VAS, visual analogue scale for ocular dryness; Oral-VAS, VAS for oral dryness; OSDI, ocular surface disease index; PGA, patient's global assessment.

Supplementary Table S3. Correlations between subjective and objective clinical disease activity indexes according to status of medication in primary Sjögren's syndrome.

	Medication (+) (N = 28)			Medication (-) (N = 38)		
	**ESSDAI**	**ClinESSDAI**	**ClinTrialsESSDAI**	**ESSDAI**	**ClinESSDAI**	**ClinTrialsESSDAI**
	Spearman rho	Spearman rho	Spearman rho	Spearman rho	Spearman rho	Spearman rho
ESSPRI	0.173	0.211	0.211	0.464**	0.496**	0.663**
ESSPRI-Dryness	0.250	0.332	0.322	0.026	0.025	0.207
ESSPRI-Fatigue	0.016	0.002	0.002	0.548**	0.516**	0.668**
ESSPRI-Pain	0.020	0.026	0.026	0.483**	0.563**	0.673**
ESS	0.302	0.361	0.361	-0.014	-0.026	0.149
Ocular-VAS	-0.015	0.122	0.122	-0.035	-0.025	0.172
Oral-VAS	0.305	0.323	0.323	-0.022	-0.058	0.107
OSDI	-0.048	-0.152	-0.152	-0.206	-0.233	-0.233
PGA	-0.091	-0.050	-0.050	0.405*	0.388*	0.518**

ESSDAI, EULAR Sjögren's Syndrome Disease Activity Index; ClinESSDAI, Clinical ESSDAI; ClintrialsESSDAI, Clinical Trials ESSDAI; ESSPRI, EULAR Sjögren's Syndrome Patient Reported Index; ESSPRI-Dryness, dryness domain of ESSPRI; ESSPRI-Fatigue, fatigue domain of ESSPRI; ESSPRI-Pain, pain domain of ESSPRI; ESS, EULAR sicca score; Ocular-VAS, visual analogue scale for ocular dryness; Oral-VAS, VAS for oral dryness; OSDI, ocular surface disease index; PGA, patient's global assessment. **P* = <0.05, ***P* = <0.01

Supplementary Table S4. Correlations between domains of ESSPRI and other PROs or objective clinical disease activity indexes in patients with primary Sjögren's syndrome.

	ESSPRI-Dryness		ESSPRI-Fatigue		ESSPRI-Pain	
	Spearman rho	*P*-value	Spearman rho	*P*-value	Spearman rho	*P*-value
ESSPRI	0.661	<0.001	0.859	<0.001	0.849	<0.001
ESSPRI-Dryness			0.360	0.003	0.376	0.002
ESSPRI-Fatigue	0.360	0.003	-	-	0.636	<0.001
ESSPRI-Pain	0.376	0.002	0.636	<0.001	-	-
ESS	0.969	<0.001	0.324	0.008	0.368	0.002
Ocular-VAS	0.799	<0.001	0.289	0.019	0.208	0.094
Oral-VAS	0.811	<0.001	0.241	0.052	0.328	0.007
OSDI	0.272	0.189	0.011	0.959	0.040	0.850
PGA	0.690	<0.001	0.527	<0.001	0.409	<0.001
ESSDAI	0.160	0.198	0.395	0.001	0.310	0.011
ClinESSDAI	0.193	0.121	0.388	0.001	0.383	0.001
ClinTrialsESSDAI	0.293	0.017	0.461	<0.001	0.440	<0.001
OSS	0.333	0.017	-0.182	0.200	-0.099	0.490
tBUT	-0.220	0.120	0.082	0.569	0.153	0.282
Unstimulated SFR (ml/min)	-0.281	0.025	0.061	0.634	-0.039	0.762
Stimulated SFR (ml/min)	-0.263	0.037	0.104	0.419	-0.033	0.797

Salivary gland scintigraphy						
PG-UR	-0.252	0.044	0.020	0.875	-0.054	0.671
PG-EF (%)	-0.227	0.071	0.136	0.283	0.097	0.448
SMG-UR	-0.357	0.004	-0.069	0.589	-0.079	0.537
SMG-EF (%)	-0.325	0.009	-0.038	0.764	-0.047	0.710
Focus score	0.261	0.050	0.006	0.962	0.086	0.525
ESR	0.156	0.210	0.365	0.003	0.142	0.256
CRP	0.133	0.289	0.430	<0.001	0.222	0.073
IgG	0.209	0.097	0.297	0.018	0.116	0.363

ESSPRI, EULAR Sjögren's Syndrome Patient Reported Index; ESSPRI-Dryness, dryness domain of ESSPRI; ESSPRI-Fatigue, fatigue domain of ESSPRI; ESSPRI-Pain, pain domain of ESSPRI; ESS, EULAR sicca score; Ocular-VAS, visual analogue scale for ocular dryness; Oral-VAS, VAS for oral dryness; OSDI, ocular surface disease index; PGA, patient's global assessment; OSS, ocular staining score; tBUT, tear break-up time; SFR, Salivary flow rate; PG, parotid gland; UR, uptake ratio; EF, ejection fraction; SMG, submandibular gland, ESR, erythrocyte sedimentation rate; CRP, C-reactive protein.

Supplementary Table S5. Clinical and laboratorial characteristics of patients with primary Sjögren's syndrome based on ESSPRI of 5.

	**ESSPRI ≥ 5** (N = 32)	**ESSPRI < 5** (N = 34)	***P*-value**
Age, years	48.2 ± 10.5	50.2 ± 12.1	0.480†
Disease duration	4.3 ± 2.8	5.9 ± 4.1	0.069
ESSPRI			
ESSPRI-Dryness	6.9 ± 1.2	5.0 ± 1.7	<0.001
ESSPRI-Fatigue	6.6 ± 1.2	3.3 ± 1.4	<0.001
ESSPRI-Pain	4.6 ± 2.1	1.6 ± 1.4	<0.001
ESS	7.2 ± 1.3	5.3 ± 1.7	<0.001
Ocular-VAS	6.1 ± 1.6	4.2 ± 2.2	<0.001
Oral-VAS	7.7 ± 1.6	5.9 ± 2.3	<0.001
OSDI	45.7 ± 27.2	37.8 ± 17.2	0.379
PGA	6.7 ± 1.5	4.3 ± 1.8	<0.001
ESSDAI	5.8 ± 6.1	2.5 ± 3.2	0.009
ClinESSDAI	5.8 ± 6.9	2.0 ± 3.5	0.007
ClintrialsESSDAI	4.5 ± 4.6	1.3 ± 2.0	0.007
Schirmer's test positivity	29/32 (90.6)	23/33 (69.7)	0.061
OSS	6.9 ± 6.4	6.9 ± 6.2	0.988
tBUT	8.9 ± 4.1	8.9 ± 4.5	0.982
Unstimulated SFR (ml/min)	0.02 ± 0.03	0.04 ± 0.08	0.392
Stimulated SFR (ml/min)	0.37 ± 0.54	0.43 ± 0.53	0.682
Salivary gland scintigraphy			
PG-UR	2.5 ± 0.9	2.5 ± 0.9	0.837
PG-EF (%)	35.9 ± 21.1	39.8 ± 21.5	0.714

SMG-UR	2.4	±	0.9	2.4	±	1.0	0.714
SMG-EF (%)	17.7	±	17.5	22.8	±	18.4	0.257
Focus score	2.2	±	2.1	1.8	±	1.6	0.448
ESR elevation	20/32		(62.5)	15/34		(44.1)	0.149‡
CRP elevation	5/32		(15.6)	0/34		(0.0)	0.023‡
IgG elevation	22/32		(68.8)	16/32		(50.0)	0.203‡
Anti-Ro antibody positivity	24/31		(77.4)	21/34		(61.8)	0.191‡
Anti-La antibody positivity	7/31		(22.6)	12/34		(35.3)	0.289‡

P values are obtained using the Student's *t*-test†, Mann–Whitney U test, or Chi-squared or Fisher's exact test‡. ESSPRI, EULAR Sjögren's Syndrome Patient Reported Index; ESSPRI-Dryness, dryness domain of ESSPRI; ESSPRI-Fatigue, fatigue domain of ESSPRI; ESSPRI-Pain, pain domain of ESSPRI; ESS, EULAR sicca score; Ocular-VAS, visual analogue scale for ocular dryness; Oral-VAS, VAS for oral dryness; OSDI, ocular surface disease index; PGA, patient's global assessment; OSS, ocular staining score; tBUT, tear break-up time; SFR, Salivary flow rate; PG, parotid gland; UR, uptake ratio; EF, ejection fraction; SMG, submandibular gland, ESR, erythrocyte sedimentation rate; CRP, C-reactive protein.

Supplementary Table S6. Correlations between age or disease duration and subjective or objective measures in primary Sjögren's syndrome.

	Age		Disease duration	
	Spearman rho	*P*-value	Spearman rho	*P*-value
ESSPRI	-0.072	0.566	-0.177	0.155
ESSPRI-Dryness	0.014	0.913	-0.004	0.977
ESSPRI-Fatigue	-0.160	0.200	-0.271	0.028
ESSPRI-Pain	-0.052	0.680	-0.136	0.275
ESS	0.028	0.826	-0.001	0.993
Ocular-VAS	-0.004	0.972	0.009	0.946
Oral-VAS	0.025	0.841	0.012	0.924
OSDI	-0.043	0.840	0.422	0.035
PGA	0.100	0.426	0.046	0.717
ESSDAI	-0.323	0.008	-0.189	0.128
ClinESSDAI	-0.208	0.094	-0.153	0.219
ClinTrialsESSDAI	-0.226	0.068	-0.101	0.417
OSS	-0.020	0.891	0.270	0.055
tBUT	-0.037	0.798	-0.276	0.050
Unstimulated SFR (ml/min)	-0.007	0.954	-0.236	0.062
Stimulated SFR (ml/min)	-0.011	0.932	-0.147	0.249
Salivary gland scintigraphy				

PG-UR	0.034	0.790	-0.203	0.108
PG-EF (%)	0.100	0.432	-0.285	0.022
SMG-UR	0.097	0.446	-0.004	0.974
SMG-EF (%)	0.102	0.423	0.039	0.761
Focal score	-0.130	0.335	-0.091	0.502
ESR	-0.298	0.015	-0.325	0.008
CRP	-0.167	0.181	-0.203	0.103
IgG	-0.414	0.001	-0.195	0.122

ESSPRI, EULAR Sjögren's Syndrome Patient Reported Index; ESSPRI-Dryness, dryness domain of ESSPRI; ESSPRI-Fatigue, fatigue domain of ESSPRI; ESSPRI-Pain, pain domain of ESSPRI; ESS, EULAR sicca score; Ocular-VAS, visual analogue scale for ocular dryness; Oral-VAS, VAS for oral dryness; OSDI, ocular surface disease index; PGA, patient's global assessment; OSS, ocular staining score; tBUT, tear break-up time; PGA, patient's global assessment; ESSDAI, EULAR Sjögren's Syndrome Disease Activity Index; ClinESSDAI, Clinical ESSDAI; ClintrialsESSDAI, Clinical Trials ESSDAI ; OSS, ocular staining score; tBUT, tear break-up time; SFR, salivary flow rate; PG, parotid gland; UR, uptake ratio; EF, ejection fraction; SMG, submandibular gland, ESR, erythrocyte sedimentation rate; CRP, C-reactive protein.
